# Six Nearly Complete Genome Segments of a Novel Reovirus Identified in Laotian Batflies

**DOI:** 10.1128/MRA.00733-19

**Published:** 2019-11-14

**Authors:** Sarah Temmam, Khamsing Vongphayloth, Jeffrey C. Hertz, Ian Sutherland, Bounsavane Douangboubpha, Marc Grandadam, Thomas Bigot, Paul T. Brey, Marc Eloit

**Affiliations:** a Institut Pasteur, Biology of Infection Unit, Inserm U1117, Pathogen Discovery Laboratory, Paris, France; b Laboratory of Vector Borne Diseases, Institut Pasteur du Laos, Vientiane, Lao People’s Democratic Republic; c United States Naval Medical Research Unit Two, Singapore; d United States Navy Entomology Center of Excellence, NAS Jacksonville, Jacksonville, Florida, USA; e Faculty of Environmental Science, Lao National University, Vientiane, Lao People’s Democratic Republic; f Arbovirology and Emerging Virus Laboratory, Institut Pasteur du Laos, Vientiane, Lao People’s Democratic Republic; g Institut Pasteur–Bioinformatics and Biostatistics Hub–Computational Biology Department, Institut Pasteur, USR 3756 CNRS, Paris, France; h National Veterinary School of Alfort, Paris-Est University, Maisons-Alfort, France; KU Leuven

## Abstract

As part of the characterization of viral communities of Laotian batflies, we report here the sequencing of six nearly complete genome segments of a novel reovirus identified in Laotian batflies that is distantly related to reoviruses recently reported in various Diptera species.

## ANNOUNCEMENT

Batflies are obligate bloodsucking Diptera (Hippoboscoidea) species belonging to two families, Streblidae and Nycteribiidae. Streblidae species have functional wings, whereas Nycteribiidae species are flattened, spiderlike flies that lack wings. Usually known as “batflies,” most species are highly host-specific and only feed on bats. Viruses belonging to the Reoviridae are double-stranded nonenveloped RNA viruses that present 9 to 12 genome segments. They have been found in many host species, from plants to vertebrate and invertebrate animals (1). Recently, six segments of two novel reoviruses in Diptera were sequenced (i.e., High Island virus in U.S. mosquitoes [2] and Hubei diptera virus 21 in a Chinese pool of Diptera species [3]). In order to characterize viral communities of bat ectoparasites in the Lao People’s Democratic Republic, we report here the sequencing of 6 nearly complete segments of a novel divergent reovirus identified in Laotian batflies.

A total of 99 batflies (8 genera belonging to the families Streblidae and Nycteribiidae) collected in 2017 in the Vientiane Province of Lao People’s Democratic Republic (Fueng, Hin Heup, and Van Vieng districts) were analyzed. Batflies were morphologically identified at the genus level. Individual specimens were suspended in 400 μl of cold phosphate-buffered saline (PBS) and crushed for 1 min at 60 Hz in a TissueLyser homogenizer (Qiagen) in the presence of Lysing Matrix E beads (MP Biomedicals). Residual tissue fragments were pelleted by spinning the tubes at 10,000 × *g* for 5 min. Half of the supernatant (200 μl) was used for total RNA purification using NucleoSpin RNA kits (Macherey-Nagel) according to the manufacturer’s instructions. The rest of the lysates were kept frozen at −80°C for viral isolation assays. Nine minipools of 11 individuals were constituted to contain both batfly families and were further used for transcriptome sequencing (RNA-Seq) library preparation using the SMARTer stranded RNA-Seq Pico Input mammalian kit v2 (Clontech). The nine libraries were sequenced in a 2 × 150-bp paired-end format on a NextSeq sequencer with the NextSeq 500/550 high-output v2 flow cell (Illumina). An average of 43 million clusters were obtained per library. An in-house bioinformatics pipeline was used, which comprised quality check and trimming (based on AlienTrimmer package v0.4.0, using default parameters except for −*p* = 80 [4]), *de novo* assembly (using MEGAHIT v1.1.2 with default parameters, except for the minimum contig length being defined as 100 bp [5]), and open reading frame (ORF) prediction (https://figshare.com/articles/translateReads_py/7588592). A BLAST-based similarity search (v2.2.26) was performed for all contigs and singletons against the protein Reference Viral Database (RVDB) (6), followed by the verification that no better hit than a virus was found when a BLAST search was performed against the whole NCBI nonredundant (nr) protein database.

Six out of nine pools of batflies included sequences related to the family *Reoviridae*. Although one pool contained only 6 singletons, which were assigned to 3 segments belonging to members of the *Reoviridae*, the other 5 pools presented large contigs with distant similarity (<65% amino acid identity) to Hubei diptera virus 21, High Island virus, Eccles virus, and Shelly Beach virus, respectively identified in Diptera, mosquitoes, *Drosophila*, and tick arthropods (Table 1). We combined reads belonging to pools BF02, BF07, and BF10 to *de novo* assemble viral segments of this novel reovirus, tentatively named Lao batfly reovirus. This virus includes at least 6 segments that present distant homologies with Hubei diptera virus 21 and High Island virus, but additional highly divergent segments may have been missed by the usual BLAST-based methods of taxonomic assignation of reads. To date, it is therefore impossible to conclusively state the number of segments comprising Lao batfly reovirus. To help with segment annotation, we aligned the 5′ and 3′ putative termini of Lao batfly reovirus with Hubei diptera virus 21 and High Island reovirus segments. Segments 4 and 6 were partially sequenced, whereas the complete ORFs were obtained for segments 1, 2, 3, and 5, with high coverage (> 500× per segment). Segment 1 encodes a protein of 1,373 amino acids (aa), and segments 2, 3, and 5 code for hypothetical proteins of 1,174, 1,076, and 595 aa, respectively (4,160 bp, 3,654 bp, 3,334 bp, and 1,898 bp, respectively, for segments 1, 2, 3, and 5). To assign a function to the segment 1 viral protein, we used the Swiss-Model program (7) to predict the tertiary structure of Lao batfly reovirus segment 1. The closest structure determined by Swiss-Model was the RNA-dependent RNA polymerase of cytoplasmic polyhedrosis virus (CPV), a reovirus that infects insects. No significant hit was obtained for other proteins coded by segments 2 to 6, leading us to propose them as hypothetical proteins. Further biochemical characterizations will help define functions for these viral proteins.

**TABLE 1:** Matrix showing the amino acid identity of Lao batfly reovirus with representative closest members of the *Reoviridae* family^*a*^

Sequence no.	Species	% amino acid identity (segment) by sequence no. and species^*b*^:										
		1 (Lao batfly reovirus)	2 (Shelly Beach virus)	3 (High Island virus)	4 (Hubei diptera virus 21)	5 (Eccles virus)	6 (Operophtera brumata reovirus)	7 (Hubei reo-like virus 3)	8 (Cimodo virus)	9 (Lutzomyia reovirus 2)	10 (Torrey Pines virus)	11 (Maize rough dwarf virus)
2	Shelly Beach virus	61.83 (S1), 57.04 (S2), 36.65 (S5)										
3	High Island virus	65.27 (S1), 54.78 (S2), 41.58 (S5)	64.89 (S1), 56.89 (S2), 42.55 (S5)									
4	Hubei diptera virus 21	55.43 (S1), 52.18 (S2), 33.19 (S5)	56.38 (S1), 50.68 (S2), 34.82 (S5)	58.10 (S1), 49.57 (S2), 35.17 (S5)								
5	Eccles virus	61.07 (S1), 51.18 (S2), 35.11 (S5)	60.69 (S1), 50.81 (S2), 37.15 (S5)	60.11 (S1), 48.94 (S2), 36.65 (S5)	66.09 (S1), 61.77 (S2), 50.64 (S5)							
6	Operophtera brumata reovirus	37.17 (S1)	37.55 (S1)	37.36 (S1)	38.87 (S1)	37.24 (S1)						
7	Hubei reo-like virus 3	22.10 (S1)	22.28 (S1)	20.97 (S1)	21.76 (S1)	21.58 (S1)	19.14 (S1)					
8	Cimodo virus	22.37 (S1)	21.62 (S1)	22.74 (S1)	22.41 (S1)	21.58 (S1)	19.81 (S1)	44.23 (S1)				
9	Lutzomyia reovirus 2	20.00 (S1)	19.43 (S1)	18.15 (S1)	19.09 (S1)	18.71 (S1)	18.16 (S1)	17.41 (S1)	17.05 (S1)			
10	Torrey Pines virus	19.62 (S1)	18.49 (S1)	20.04 (S1)	20.60 (S1)	18.71 (S1)	16.54 (S1)	20.00 (S1)	20.08 (S1)	32.19 (S1)		
11	Maize rough dwarf virus	21.38 (S1)	20.63 (S1)	21.56 (S1)	20.19 (S1)	19.81 (S1)	18.73 (S1)	21.59 (S1)	19.96 (S1)	15.62 (S1)	17.18 (S1)	
12	Rice black streaked dwarf virus	21.19 (S1)	21.00 (S1)	21.56 (S1)	20.19 (S1)	19.44 (S1)	18.35 (S1)	21.40 (S1)	19.96 (S1)	15.81 (S1)	17.37 (S1)	96.46 (S1)

a Accession numbers are given here for segments 1, 2, and 5, respectively, for viruses numbered 1 to 5, and for segment 1 for viruses numbered 6 to 12. Sequence numbers, species, and accession numbers are as follows: 1, Lao batfly reovirus (GenBank accession numbers QBA09477, QBA09478, and QBA09481); 2, Shelly Beach virus (AYP67577, AYP67578, and AYP67581); 3, High Island virus (AVO64750, MF094129 [translation in amino acids of the nucleotide sequence], and AVO64753); 4, Hubei diptera virus 21 (APG79176, APG79177, and APG79180); 5, Eccles virus (AWA82237, AWA82238, and AWA82241); 6, Operophtera brumata reovirus (YP_392501); 7, Hubei reo-like virus 3 (APG79172); 8, Cimodo virus (AHF20715); 9, Lutzomyia reovirus 2 (AKP18622); 10, Torrey Pines virus (AWY11145); 11, Maize rough dwarf virus (ANG56321); and 12, Rice black streaked dwarf virus (AOS58315).

b S1 refers to segment 1, coding for the RNA-dependent RNA polymerase; S2 refers to segment 2 complete coding sequence (CDS); S5 refers to segment 5 complete CDS.

The bat study was approved by the Institutional Animal Care and Use Committee (IACUC) of the Department of Forest Resource Management (DFRM). For the Lao People’s Democratic Republic side, the authorization agreement for carrying out bat capture was approved by the wildlife authorities of the DFRM and by the Ministry of Agriculture and Forestry, Lao People’s Democratic Republic (approval number 0174/DFRM, issued January 2017).

### Data availability.

The genome sequence of Lao batfly reovirus strain BF02/7/10 was deposited in GenBank under accession numbers MK468721 to MK468726. Raw data corresponding to Lao batfly reovirus were deposited in the SRA database under accession number SRR8592028.
